# The Bane of Influence in Phthisis Work

**Published:** 1913-10-04

**Authors:** 


### EDITOR'S LETTER-BOX.

### The Bane of Influence in Phthisis Work.

*To the Editor of* The Hospital.

Sir,—In reference to your note on "The bane of influence in phthisis work," would it not be well to suggest that matrons appointed to start sanatoria ought to have several years' experience in sanatorium work? I knew of a case this week where a matron was appointed to start a new sanatorium without any experience in sanatorium work. I do not wish my name made public, but I shall be glad if you can point out the desirability of matrons having sanatorium experience—as well as doctors.—Yours faithfully,

Sanatorium Matron.

September 26, 1913.

